# The Correlation Between Smoking and Severity of COVID-19 Infection in Tribal Populations: A Single-Center Retrospective Cross-Sectional Study

**DOI:** 10.7759/cureus.99093

**Published:** 2025-12-13

**Authors:** Aarsh Sathia, Vikrant Ghadge, Ruchi Rana, Yash Parpani, Devarsh Bhirud, Vaishnavi Raut

**Affiliations:** 1 General Surgery, Bharatratna Dr Babasaheb Ambedkar Municipal General Hospital, Mumbai, IND; 2 Internal Medicine, Surrey and Sussex Healthcare NHS Trust, Redhill, GBR; 3 General Surgery, Oscar Multispeciality Hospital, Mumbai, IND; 4 Medicine, Topiwala National Medical College and BYL Nair Charitable Hospital, Mumbai, IND; 5 General Surgery, NC Medical College, Haryana, IND; 6 Medicine, Subdistrict Hospital Teosa, Amravati, IND

**Keywords:** covid 19, ct severity score (ctss), public health, smoking, tribal population

## Abstract

Background

Cigarette smoking is a well-established risk factor for respiratory diseases and has been proposed to worsen outcomes in COVID-19 infection. Limited evidence exists regarding this association among tribal populations in India, who often face socioeconomic and healthcare disparities. This study aimed to assess the correlation between cigarette smoking and the severity of COVID-19 infection among the tribal population of North Maharashtra.

Methods

A retrospective cross-sectional study was conducted among 75 patients with a confirmed history of COVID-19 infection in the tribal areas of Dhule District, Maharashtra, during July 2021. Data were collected through retrospective review of outpatient and inpatient medical records. Demographic details, smoking status, duration and intensity of smoking, comorbidities, and COVID-19 severity indicators were recorded. Associations between smoking variables and disease severity parameters, including CT severity score, ventilator requirement, breathlessness, and oxygen requirement, were analyzed using the Chi-square (χ²) test. A p-value <0.05 was considered statistically significant.

Results

Of the total (75) participants, 43 (57.3%) were smokers and 32 (42.7%) were non-smokers. A significant positive association was observed between daily cigarette consumption and CT severity score (χ²=50.406, p<0.001), and between duration of smoking and ventilator requirement (χ²=14.540, p=0.006). Longer smoking duration was also significantly associated with breathlessness as a presenting complaint (χ²=11.223, p=0.024). No statistically significant association was found between smoking status and oxygen requirement (p=0.077).

Conclusion

The findings indicate that cigarette smoking is significantly associated with increased disease severity and respiratory complications in COVID-19 among tribal populations. Individuals with a longer smoking history demonstrated higher CT severity scores and a greater need for ventilatory support. These results underscore the importance of targeted smoking cessation and respiratory health initiatives within vulnerable tribal communities to mitigate future pandemic-related risks.

## Introduction

Severe acute respiratory syndrome coronavirus 2 (SARS-CoV-2) causes Coronavirus disease 2019 (COVID-19), which is evidently a highly contagious disease and a systemic disease with a major respiratory component. The major mode of transmission is by airborne droplets or by aerosols and person-to-person contact. In the respiratory tract, COVID-19 has an affinity for lung epithelial cells as they have angiotensin-converting enzyme 2 (ACE2) receptors to which the viral spike glycoprotein binds, causing viral pneumonia and acute respiratory distress syndrome. The individuals prone to higher rates of complications and mortality due to COVID-19 are older than 60 years, with comorbidities like diabetes mellitus, hypertension, chronic lung disease (CLD), and immunosuppressed individuals. Some studies have suggested that infection with SARS-CoV-2 is associated with other risk factors, such as smoking, external environmental pollution, and certain climatic conditions [1]. The aim of this study is to evaluate the association between cigarette smoking and the severity of COVID-19 infection in tribal populations.

Coronaviruses are RNA viruses that can be classified into four genera based on their genomic characteristics: α, β, π, and ξ [2]. Based on sequencing and evolutionary data, bats are the currently proposed source of reservoir coronaviruses [3,4]. COVID-19 infection has a high homology (>80%) to severe acute respiratory syndrome coronavirus (SARS-CoV), which was responsible for the acute respiratory distress syndrome (ARDS) outbreak in Guangdong Province in Southeast China in 2003. The World Health Organization (WHO) declared that the COVID-19 outbreak is a pandemic on 11 March 2020 [5]. According to the WHO and Centers for Disease Control and Prevention (CDC), this virus commonly spreads due to respiratory droplets produced during sneezing and coughing. The virus invades the body through exposed mucosal surfaces, such as the nasal, oral, and upper respiratory tract, and, less frequently, through the conjunctival mucosa. Commonly, it includes symptoms like breathlessness, generalized weakness, fever, cough, malaise, anosmia, and ageusia. Other less frequent symptoms include headaches, loss of appetite, myalgia, and diarrhea.

Smoking is a well-established risk factor in various respiratory diseases, for example, lung cancer, chronic obstructive pulmonary disease (COPD), and asthma. Hence, an attempt can be made to link smoking with COVID-19, which also has a major respiratory component. According to the latest studies, a more severe form of COVID-19 is seen in patients with a higher number of comorbidities as opposed to healthy individuals having fewer comorbidities. SARS-CoV-2 is likely similar to avian influenza H7N9 and SARS-CoV infections, which are readily predisposed to cause respiratory failure and death in susceptible patients having comorbidities like hypertension, diabetes, cancer, and cardiovascular diseases. Every year, more than eight million deaths are associated with tobacco consumption globally [6]. It is a known fact that smokers experience more respiratory complications than non-smokers.

Smoking can cause upregulation of the ACE2 receptor, the receptor known for both the SARS-CoV and the human respiratory coronavirus NL638 [7]. In SARS-CoV-2, host cells are recognised by the spike protein on the surface of the viral envelope. The spike protein binds to the ACE2 receptor protein in cells. After this, the spike protein can be cleaved by the serine protease TMPRSS2. The fusion of the viral envelope with the membranes in the host cell allows for viral entry into the cell. This is vital for the entry of SARS-CoV-2 into human host cells, and thus it plays an integral role in COVID-19 infection and disease progression [8]. Zhao et al. [9] observed that ACE2 is expressed explicitly in type-2 pneumocytes, in which the genes that regulate viral reproduction and transmission are highly expressed. The dysregulation of the renin-angiotensin system (RAS) pathway by the coronavirus occurs through the sequestration of ACE2, contributing to increased morbidity [10]. Disease progression is influenced by ACE2 levels; in a study using mice engineered to express human ACE2, those with the highest ACE2 mRNA levels had the shortest survival time following exposure to the coronavirus [11]. This gives the indication that smokers may be more susceptible to infection by SARS-CoV-2 and possibly COVID-19.

The health struggles confronted by indigenous communities stem from their social and physical surroundings. Factors like limited income, restricted access to education and employment, insufficient healthcare, food insecurity, substandard housing, and the absence of proper safety nets contribute to their health disparities [12]. The enduring impact of colonization suppresses indigenous values and cultural practices, exacerbating wellbeing challenges. Moreover, residing in regions prone to climate extremes and long-term changes heightens the vulnerability to COVID-19 transmission, compounding the health risks faced by these communities [13].

Smokers also experience increased rates of influenza, bacterial pneumonia, and tuberculosis [14]. As mentioned in previous studies, patients with COPD are more susceptible to viral infections and present worse outcomes. Notably, patients with pre-existing COPD and current tobacco use are much more likely to experience poor therapeutic outcomes from COVID-19 [15]. Patients having a history of smoking are more predisposed to COVID-19 compared to non-smokers, and are associated with higher rates of admission to intensive care units (ICU), use of ventilators, and death. As multiple respiratory infections imply smoking is a major risk factor due to its immunocompromising effects, an attempt can be made to link smoking with COVID-19, which also has a major respiratory component. The WHO confirmed that smokers may experience more severe complications from COVID-19 than non-smokers [6].

The study was conducted in July 2021, during the post-second-wave period of the COVID-19 pandemic in India, when the tribal regions of Dhule experienced a surge in cases but remained underserved in terms of diagnostic and treatment facilities. This setting was selected due to its high proportion of tobacco users and limited healthcare access, making it an important population for evaluating risk factors associated with COVID-19 severity. Considering the complications resulting from smoking in patients with viral infections, the primary aim of this research is to attempt to find an association between smoking and COVID-19 outcomes and severity.

This study primarily aimed to evaluate the association between cigarette smoking and the severity of COVID-19 infection in the tribal population of Dhule. Additionally, the study sought to compare clinical outcomes such as CT severity score and ventilator requirement between smokers and non-smokers, to assess how smoking duration and daily cigarette consumption relate to respiratory symptoms, including breathlessness, and to examine the influence of pre-existing comorbidities on COVID-19 severity among smokers.

## Materials and methods

A study was conducted in the tribal areas of Dhule in July 2021. A convenience sampling approach was used. All outpatient and inpatient medical records from patients attending the Annasaheb Chudaman Patil Memorial (ACPM) Medical College Hospital between July 1 and July 31, 2021, were reviewed consecutively, and all eligible cases meeting the inclusion criteria were included. Their medical records were assessed for data collection. COVID-19 infection was confirmed based on documented RT-PCR test results in the medical records. Antigen-positive cases were included only if subsequently verified by RT-PCR. Clinical diagnosis without laboratory confirmation was not accepted for inclusion. Given the cultural diversity of these regions and the high prevalence of tobacco use, we sought to examine the association between cigarette smoking and the severity of COVID-19 infection in the tribal population. Furthermore, we investigated the association between smoking prevalence, comorbidities, and COVID-19. The study included 75 participants who were screened according to the following criteria.

Participants included in the study were individuals above 18 years of age with a confirmed history of COVID-19 infection. Both smokers and non-smokers were eligible for inclusion. Individuals younger than 18 years of age and those without a documented history of COVID-19 infection were excluded from the study.

All data were anonymized and entered into a secure spreadsheet using Microsoft Excel (Microsoft Corp., Redmond, US). Descriptive statistics were used to summarize demographic characteristics and smoking-related variables. Categorical variables such as smoking status, duration of smoking, daily cigarette consumption, ventilator requirement, and oxygen requirement were presented as frequencies and percentages. Smoking exposure was standardized using cigarette equivalents. One pack was defined as 10 cigarettes or 10 bidis. Daily smoking was categorized as packs/day. Mixed tobacco users were classified based on their combined total cigarette-equivalent consumption. Duration of smoking was recorded in years. Records with missing information for key variables (smoking exposure, CT severity score, ventilator requirement, or oxygen requirement) were excluded from the corresponding analyses. No data imputation methods were applied.

Comparative analyses were performed to explore associations between smoking variables and COVID-19 severity indicators, including CT severity score, ventilator requirement, breathlessness, and oxygen requirement. The Chi-square (χ²) test of independence was employed to determine the significance of associations between categorical variables. A p-value of <0.05 was considered statistically significant. All collected data were entered and analyzed using SPSS V.27 (trial version; IBM Corp., Armonk, US). The CT severity score (CTSS) was used to quantify the extent of pulmonary involvement in COVID-19 infection. The scoring system was adapted from the method described by Pan et al. [16]. The CTSS was assessed using the 25-point scoring system. Each of the five lung lobes was visually evaluated and assigned a score from 0 to 5 based on the percentage of involvement, resulting in a total score ranging from 0 to 25, with higher scores indicating greater pulmonary involvement.

Consent forms were prepared in English and local languages (Hindi and Marathi), and informed consent was obtained from all participants prior to data collection. The study was conducted as a retrospective audit and review of outpatient and inpatient medical records, with no direct patient intervention or interaction. All data were collected anonymously, and issues of confidentiality and privacy were strictly maintained throughout the study. Patient identifiers were excluded from the dataset, and information was anonymized prior to analysis. The institution reviewed and provided a letter confirming the anonymity of patient data and approval for the study.

## Results

**Figure 1:**
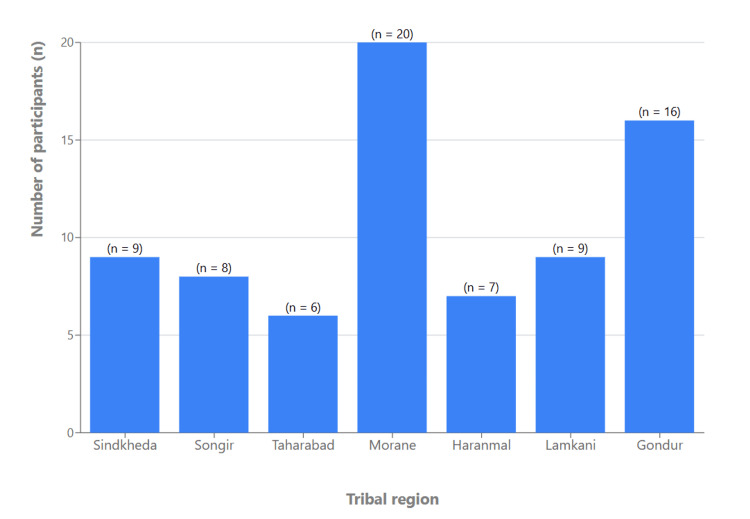
Distribution of study participants according to geographic region in the tribal areas

**Figure 2:**
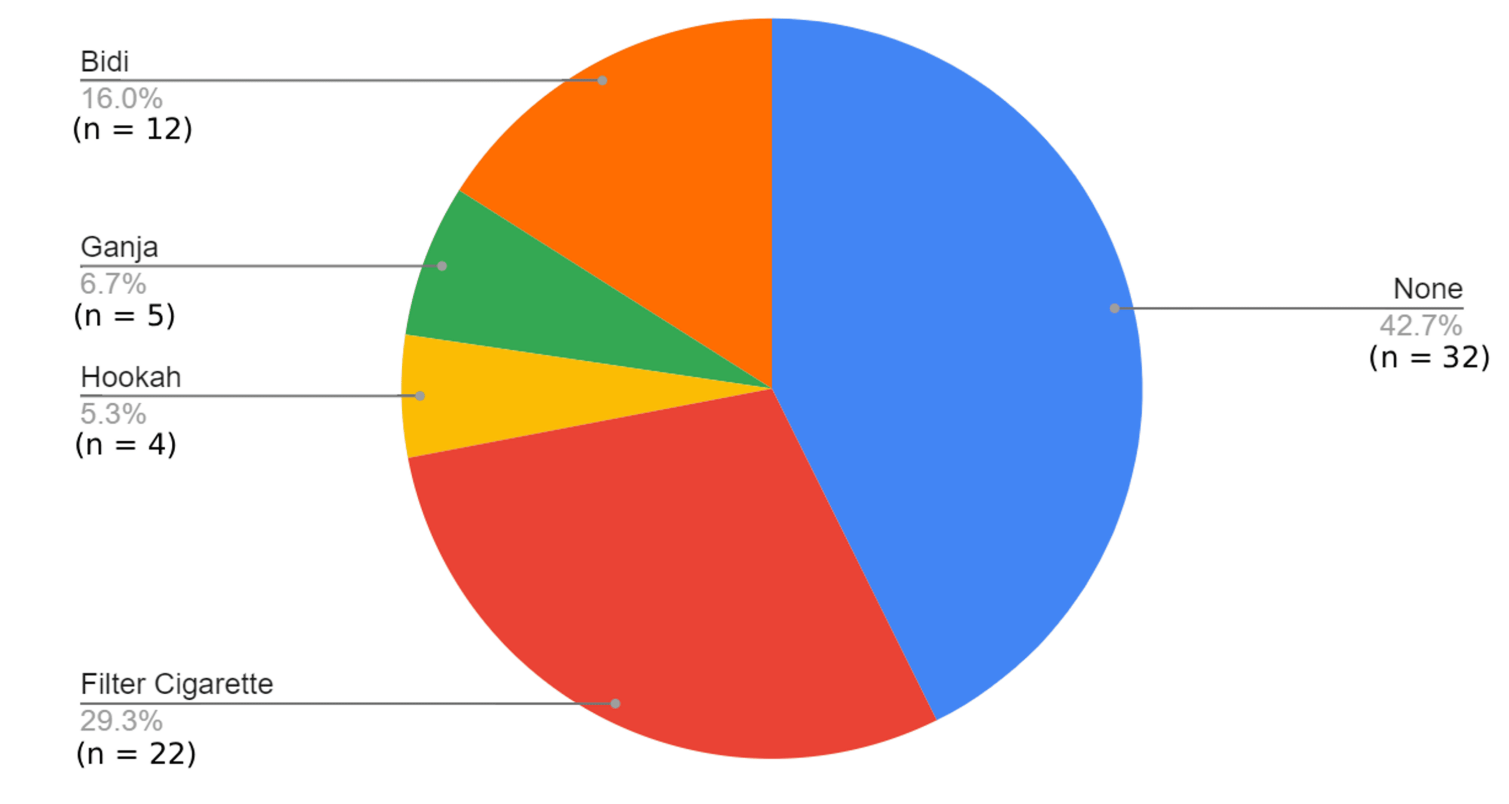
Types of smoking and substance use among study participants

**Figure 3:**
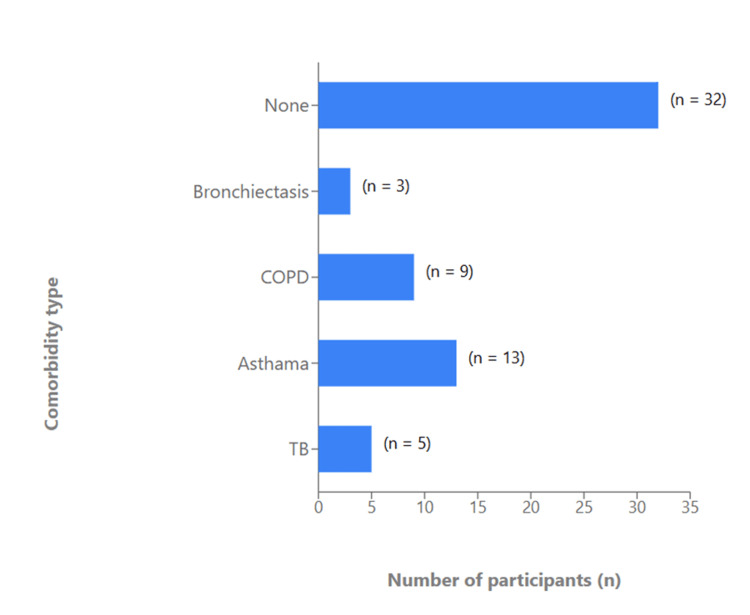
Prevalence of comorbidities among study participants.

Participants were drawn from multiple tribal regions of Dhule district, with the highest representation from Morane (Figure 1). Filter cigarette smoking was the most common form of tobacco use (n=22, 29.3%), followed by bidi smoking (n=12, 16.0%), while 42.7% (n=32) of participants reported no smoking habit. Smaller proportions used ganja (n=5, 6.7%) or hookah (n=4, 5.3%; see Figure 2). Asthma (n=13, 17.3%) and COPD (n=9, 12.0%) were the most frequent respiratory comorbidities identified, followed by tuberculosis (n=5, 6.7%) and bronchiectasis (n=3, 4.0%). Nearly half of the participants (n=32, 42.7%) reported no underlying respiratory illness (Figure 3).

Out of 75 participants, 43 (57.3%) were smokers, and 32 (42.7%) were non-smokers, indicating a higher prevalence of smoking within the study population (Table 1). Among smokers, most participants (n=21, 28.0%) reported a smoking history of 5-10 years, while 18.7% (n=14) had smoked for 10-20 years. Only 6.7% (n=5) reported smoking for more than 20 years (Table 2). A majority of smokers (n=19, 25.3%) consumed approximately two packs of cigarettes per day, while 16.0% (n=12) smoked less than one pack daily. Heavy smoking (>three packs per day) was reported by 6.7% (n=5) of participants (Table 3).

**Table 1:** Smoking status of study participants

Smoking status	Frequency	Percentage
Smokers	43	57.3
Non-smokers	32	42.7

**Table 2:** Duration of smoking among study participants

Duration of smoking	Frequency	Percentage
<5 years	3	4
>20 years	5	6.7
10-20 years	14	18.7
5-10 years	21	28
None	32	42.7
Total	75	100

**Table 3:** Daily cigarette consumption among study participants

Daily cigarette consumption	Frequency	Percent
<1 pack	12	16
>3 packs	5	6.7
2 packs	19	25.3
3 packs	7	9.3
None	32	42.7
Total	75	100

The present study demonstrated a statistically significant positive association between the number of cigarettes smoked and the CT severity score among patients following COVID-19 infection (χ²=50.406, df=16, p<0.001; Table 4). Furthermore, the duration of smoking, expressed as the number of years smoked, exhibited a strong positive correlation with the severity of COVID-19 illness. A significant association was also observed between the duration of smoking and the requirement for ventilatory support (χ²=14.540, df=4, p=0.006; Table 5), indicating that individuals with prolonged smoking histories were more likely to necessitate advanced respiratory interventions.

**Table 4:** Association between daily cigarette consumption and CT severity score df - degrees of freedom

Chi-square test results	Value	df	p-value
Pearson Chi-square	50.406	16	<0.001
Likelihood ratio	39.447	16	0.001
N of valid cases	75		

**Table 5:** Association between smoking duration and ventilator requirement among COVID-19 patients df - degrees of freedom

Chi-square test results	Value	df	p-value
Pearson Chi-square	14.540	4	0.006
Likelihood ratio	10.757	4	0.029
N of valid cases	75		

Additionally, a significant positive association was identified between the duration of smoking and the presence of breathlessness as a presenting complaint (χ²=11.223, df=4, p=0.024; Table 6). This finding suggests that chronic smoking may exacerbate respiratory symptoms, contributing to persistent dyspnea even following recovery from COVID-19 infection.

**Table 6:** Association between smoking duration and breathlessness among COVID-19 patients df - degrees of freedom

Chi-square test results	Value	df	p-value
Pearson Chi-square	11.223	4	0.024
Likelihood ratio	12.562	4	0.014
N of valid cases	75		

In contrast, no significant association was found between smoking status and the requirement for supplemental oxygen (χ²=3.127, df=1, p=0.077; Table 7), suggesting that oxygen dependency was independent of smoking as an isolated risk factor. Nevertheless, a positive association was noted between the duration of smoking and the type or intensity of treatment required for COVID-19, implying that patients with longer smoking histories tended to require more intensive therapeutic interventions.

**Table 7:** Association between smoking status and oxygen requirement during COVID-19 treatment df - degrees of freedom

Chi-square test results	Value	df	p-value
Pearson chi-square	3.127	1	0.077
Continuity correction	2.356	1	0.125
Likelihood ratio	3.153	1	0.076
N of valid cases	75		

## Discussion

This study aimed to evaluate the association between cigarette smoking and the severity of COVID-19 infection among the tribal population of North Maharashtra. The findings revealed a significant relationship between smoking and greater disease severity, reflected by higher CT severity scores and increased ventilator requirements among smokers compared to non-smokers. These results are consistent with the findings of Gülsen et al. [17], who observed a higher prevalence of smoking among severe COVID-19 cases, and Patanavanich and Glantz [18], who reported that smokers had nearly twice the odds of disease progression compared to non-smokers. Such evidence supports the hypothesis that smoking acts as a significant modifiable risk factor influencing COVID-19 severity.

However, previous studies have reported conflicting results, showing no significant association between smoking history and COVID-19 outcomes [19-23]. These inconsistencies may reflect variations in study populations, definitions of smoking, and differences in comorbidity profiles. In the present study, the predominance of male smokers and the frequent use of filter cigarettes and bidis-likely influenced by socioeconomic factors-reflect unique cultural and economic patterns within the tribal community. Smoking is well known to cause chronic structural and functional damage to the respiratory system, predisposing individuals to infections such as COVID-19. The high prevalence of respiratory comorbidities, particularly asthma and COPD, among smokers in this study may have contributed to more severe disease presentations. Additionally, other observed comorbidities such as diabetes, hypertension, and cardiovascular disease further increase the risk of adverse outcomes.

Overall, the findings emphasize that smoking independently contributes to the progression and severity of COVID-19, even in individuals without major comorbidities. However, given the cross-sectional and retrospective nature of this study, these associations should not be interpreted as causal. It is not possible to determine whether smoking directly contributes to more severe COVID-19 outcomes; rather, our results indicate that smoking patterns are correlated with indicators of disease severity in this cohort. Further longitudinal research is needed to explore causal pathways and to adjust for additional confounding factors. Given the compounded vulnerability of tribal populations, owing to limited healthcare access and socioeconomic disparities, targeted smoking cessation initiatives and community-level preventive measures are crucial to mitigate the health burden associated with both smoking and infectious respiratory diseases.

Limitations

This study has limitations that should be considered when interpreting the findings. First, the sample size was relatively small (n=75) and derived from a single center, which may limit the generalizability of the results to broader tribal or non-tribal populations. The retrospective design relied on previously recorded clinical data, and smoking history was self-reported, introducing the possibility of recall or reporting bias. Although key smoking variables such as duration and daily consumption were recorded, biochemical verification was not feasible. Additionally, the study did not control for potential confounders such as socioeconomic status, vaccination status, baseline pulmonary function, or other environmental exposures, which may have influenced both smoking behavior and COVID-19 severity. Finally, because the study was conducted during a specific one-month period (July 2021), the findings may not fully reflect patterns observed at other stages of the pandemic. Despite these limitations, the study provides valuable insights into smoking-related risks among an underserved tribal population and highlights areas for future research.

## Conclusions

The study demonstrates a significant association between cigarette smoking and the severity of COVID-19 infection. Individuals with a longer duration of smoking and higher daily cigarette consumption exhibited increased CT severity scores, a higher likelihood of requiring ventilatory support, and a greater prevalence of breathlessness as a presenting symptom. These findings indicate that chronic smoking not only predisposes individuals to more severe forms of COVID-19 but may also contribute to persistent post-recovery respiratory symptoms. Although no statistically significant association was found between smoking status and oxygen requirement, the overall pattern suggests that prolonged smoking history correlates with more intensive treatment needs and poorer clinical outcomes. The observed trends are consistent with existing evidence linking smoking-induced upregulation of ACE2 receptors to increased susceptibility and disease severity in COVID-19.

Importantly, this study highlights that the tribal population, already facing socio-economic and healthcare disparities, may experience compounded risks from smoking-related respiratory vulnerability during pandemics such as COVID-19. While these associations are clinically meaningful, the cross-sectional design limits causal interpretation. The findings highlight the need for targeted public health interventions and future prospective studies to better understand the relationship between smoking and COVID-19 severity in underserved populations.
